# Prediction of clinically relevant Pancreatico-enteric Anastomotic Fistulas after Pancreatoduodenectomy using deep learning of Preoperative Computed Tomography

**DOI:** 10.7150/thno.49671

**Published:** 2020-08-01

**Authors:** Wei Mu, Chang Liu, Feng Gao, Yafei Qi, Hong Lu, Zaiyi Liu, Xianyi Zhang, Xiaoli Cai, Ruo Yun Ji, Yang Hou, Jie Tian, Yu Shi

**Affiliations:** 1 Beijing Advanced Innovation Center for Big Data-Based Precision Medicine, School of Medicine, Beihang University, Beijing, China, 100191.; 2 Department of Radiology, Shengjing Hospital of China Medical University, Shenyang, China.; 3 Department of Pancreato-thyroidic Surgery, Shengjing Hospital of China Medical University, Shenyang, China.; 4 Department of Pathology, Shengjing Hospital of China Medical University, Shenyang, China.; 5 Department of Radiology, Tianjin Medical University Cancer Institute and Hospital, National Clinical Research Center of Cancer, Key Laboratory of Cancer Prevention and Therapy, Tianjin, PR China.; 6 Department of Radiology, Guangdong General Hospital, Guangdong Academy of Medical Sciences, 106 Zhongshan Er Road, Guangzhou 510080, China.; 7 Key Laboratory of Molecular Imaging, Chinese Academy of Sciences, Beijing, China, 100190.

**Keywords:** Pancreatic fistula, fistula risk score, pancreatoduodenectomy, computed tomography (CT), deep learning

## Abstract

**Rationale:** Clinically relevant postoperative pancreatic fistula (CR-POPF) is among the most formidable complications after pancreatoduodenectomy (PD), heightening morbidity/mortality rates. Fistula Risk Score (FRS) is a well-developed predictor, but it is an intraoperative predictor and quantifies >50% patients as intermediate risk. Therefore, an accurate and easy-to-use preoperative index is desired. Herein, we test the hypothesis that quantitative analysis of contrast-enhanced computed tomography (CE-CT) with deep learning could predict CR-POPFs.

**Methods:** A group of 513 patients underwent pancreatico-enteric anastomosis after PD at three institutions between 2006 and 2019 was retrospectively collected, and formed a training (70%) and a validation dataset (30%) randomly. A convolutional neural network was trained and generated a deep-learning score (DLS) to identify the patients with higher risk of CR-POPF preoperatively using CE-CT images, which was further externally tested in a prospective cohort collected from August 2018 to June 2019 at the fourth institution. The biological underpinnings of DLS were assessed using histomorphological data by multivariate linear regression analysis.

**Results:** CR-POPFs developed in 95 patients (16.3%) in total. Compared to FRS, the DLS offered significantly greater predictability in training (AUC:0.85 [95% CI, 0.80-0.90] *vs.* 0.78 [95% CI, 0.72-0.84]; *P* = 0.03), validation (0.81 [95% CI, 0.72-0.89] *vs.* 0.76 [95% CI, 0.66-0.84], *P* = 0.05) and test (0.89 [95% CI, 0.79-0.96] *vs.* 0.73 [95% CI, 0.61-0.83], *P* < 0.001) cohorts. Especially in the challenging patients of intermediate risk (FRS: 3-6), the DLS showed significantly higher accuracy (training: 79.9% *vs.* 61.5% [*P* = 0.005]; validation: 70.3% *vs.* 56.3% [*P* = 0.04]; test: 92.1% *vs.* 65.8% [*P* = 0.013]). Additionally, DLS was independently associated with pancreatic fibrosis (coefficients: -0.167), main pancreatic duct (coefficients: -0.445) and remnant volume (coefficients: 0.138) in multivariate linear regression analysis (r^2^ = 0.512, *P* < 0.001). The user satisfaction score in the test cohort was 4 out of 5.

**Conclusions:** Preoperative CT based deep-learning model provides a promising novel method for predicting CR-POPF occurrences after PD, especially at intermediate FRS risk level. This has a potential to be integrated into radiologic reporting system or incorporated into surgical planning software to accommodate the preferences of surgeons to optimize preoperative strategies, intraoperative decision-making, and even postoperative care.

## Introduction

Postoperative pancreatic fistula (POPF) is among the most formidable complications after pancreatoduodenectomy (PD), threatening to prolong hospitalization, increasing medical treatment costs, imposing catastrophic abscess or hemorrhage, and heightening morbidity/mortality rates 1-8. Surgical and technologic advancements have dramatically reduced procedure-related mortality, but rates of clinically relevant postoperative pancreatic fistula (CR-POPF) have remained unchanged (~11-15%) 3. Managing CR-POPF is thus a high-priority issue for pancreatic surgeons, who need sensitive predictors of CR-POPF to identify high-risk patients and adjust intra- or postoperative care accordingly.

The reported risk factors for CR-POPFs are broadly classifiable as local factors at pancreatic remnant 9, systemic factors (*e.g*., high body mass index [BMI]), and operative factors (eg, blood loss) 10-13. Local risk factors at pancreatic remnant, which strongly linked to underlying local histopathologic changes, such as rich viable gland, absence of fibrosis 14, and fatty pancreas 15, 16, represent the most likely determinants directly related to anatomic failure. The Fistula Risk Score (FRS), which incorporated four of the aforementioned parameters: small-sized main pancreatic duct (MPD), soft glandular texture (by surgeon's palpation), high-risk pathology (chronic pancreatitis [CP] or pancreatic adenocarcinoma [PDAC]), and undue intraoperative blood loss, is a well-developed and validated 10-point scale used to intraoperatively predict CR-POPF development after PD 17-19. Despite the simplicity and convenience of the FRS, it relies on subjective intraoperative findings of surgeons. Moreover, >50% patients qualify as intermediate risk (FRS scores of 3-6), which is a grey zone warranting more objective and reliable predictors.

Beyond FRS, local risk factors via quantitative imaging from standard-of-care contrast-enhanced computed tomography (CE-CT) images, such as pancreatic thickness 20 or remnant volume 21, 22, have been measured manually in dozens of studies conducted during the last decade, showing promising results in predicting CR-POPF events. Recently, the artificial intelligence (AI) based medical image analysis provides an objective and automatic way to capture all important local properties due to its self-learning characteristics, and has achieved success in different fields 23-26. However, deep-learning model hasn't been investigated in CR-POPF prediction.

Thus, the purpose of this study was to (1) develop and validate an easy-to-use deep-learning model to predict CR-POPF after PD, and (2) determine its diagnostic performance and compare it with FRS, and finally (3) investigate the histomorphologic changes pertaining to deep-learning score (DLS) generated by the deep-learning model.

## Methods

### Study population

**Figure 1:**
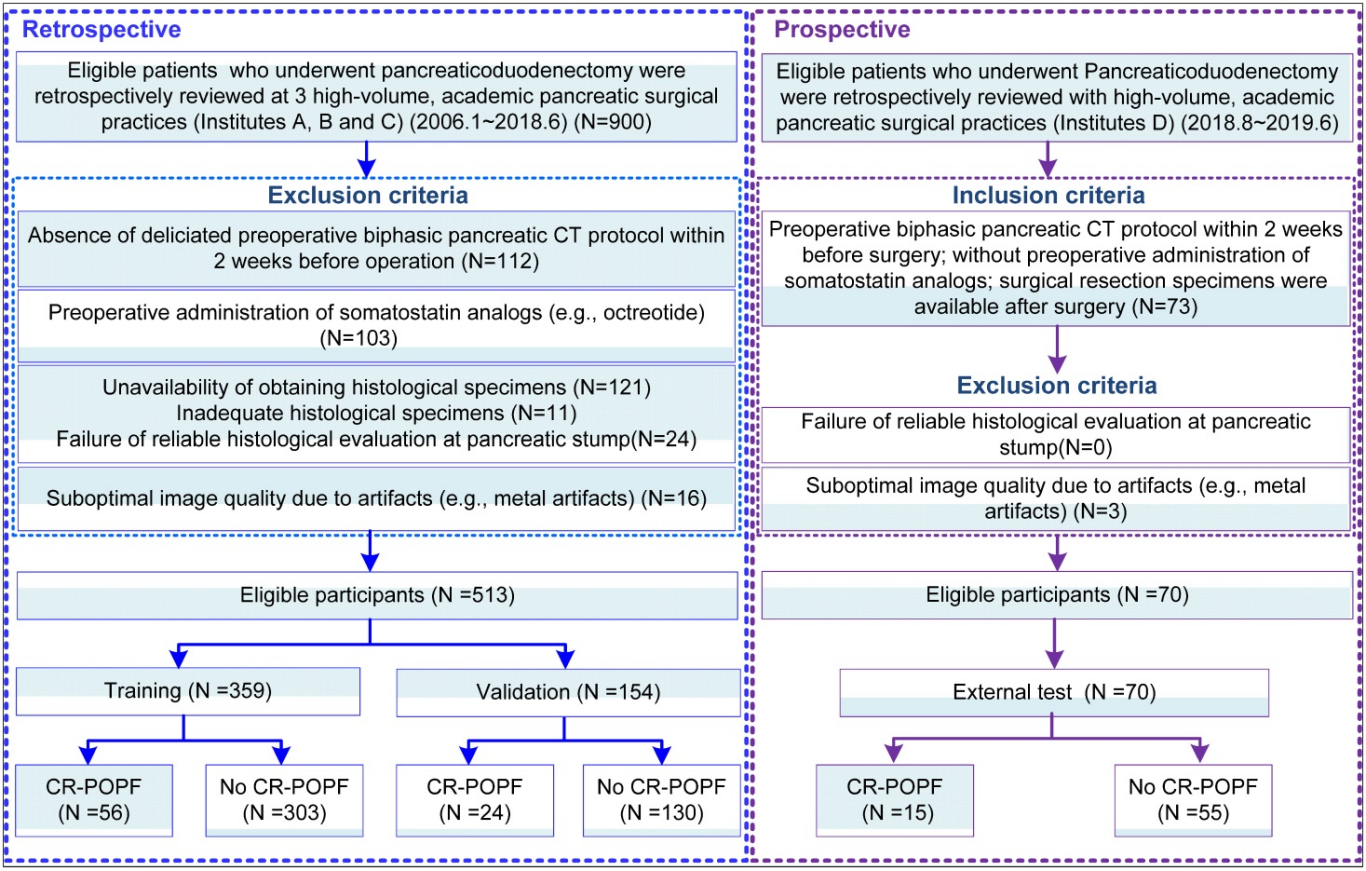
** Schematic of study design:** Data from Institutes A, B and C pertained to clinical information and corresponding imaging details, serving to train and validate the deep learning signature (DLS). Clinical and imaging data contributed by Institute D was used for external test.

This was a diagnostic, multicenter, multi-cohort study involving four cohorts from four high-volume academic institutions. This study was approved by the Institutional Review Board of each local institution and adhered to ethical standards of the 1964 Helsinki Declaration, including its later amendments. Informed consent was waived in all cohorts. From institutions A-C, 513 patients were obtained retrospectively from radiologic and pathologic archives, and were randomly divided into a training (n = 359) and validation cohort (n = 154) randomly with a ratio of 70/30 to train and validate a deep-learning model based on preoperative CE-CT images. Another cohort (Institution D) was prospectively collected as the external test (n = 70) dataset, all summarized in **Figure 1**. Notably, for this prospective cohort, patients who were eligible for pancreaticoduodenectomy were consecutively collected during the period. The radiologists who interpreted the radiological images were blinded to the clinical and laboratory results. The surgeons were also blinded to the estimated POPF risk by the deep-learning model. STROBE guidelines 27 for reporting observational studies were applied during study design, training, validation, and reporting of the prediction model.

### Clinical data collection and mitigation strategies

Five lead pancreatic surgeons (>20 years of collective pancreatic surgical experience) performed all PDs in conjunction with either pancreaticojejunostomy (PJ) or pancreaticogastrostomy (PG) 28 for a full array of indications. Medical records provided demographic and clinical data, including age, sex, BMI, diabetes mellitus, reported weight loss in the previous 6 months, jaundice, smoking, or alcohol abuse. POPFs were graded in accordance with ISGPF standards 29, 30 as either biochemical or clinically relevant (see **Supplemental Table S1)**.

The four risk factors required for FRS calculations (see **Supplemental Table S2**) were obtained from operative notes retrospectively or prospectively recorded during surgery by attending surgeons attuned to this study. These risk factors served to generate FRS scores (0-10) individually and thus categorized fistula risk as low (0-2 points), intermediate (3-6 points), or high (7-10 points). Other details are included as **Supplemental material S1.**

For fistula mitigation, less than 1% of patients received biologic sealants; two laminar intraperitoneal drains were routinely placed (100%), and 52 (10%) patients retained an additional retroperitoneal drain. Postoperative somatostatin analogs (*eg*, prophylactic octreotide) were administered to 225 (43.9%) patients, and trans-anastomotic stents (largely short, internal silicone elastomer tubes) were deployed in 281 (54.8%) patients.

### Histology of pancreatic stump

Specimens of the pancreatic stump were evaluated to quantify fibrous tissue, exocrine glandular atrophy (A) 31 and degrees of lipomatosis (L) 32, 33, as detailed **Supplemental material S2**.

### Preoperative CT imaging and segmentation

CT scanning parameters and detailed descriptions are presented in **Supplemental Table S3.** All patients underwent preoperative multiphasic scans (64-channel multi-detector CT or better) within 4 weeks of surgery at <3-mm minimum slice thickness and in three standard-of-care phases, adhering to current National Comprehensive Cancer Network (NCCN) guidelines 34, 35. A nonionic contrast agent containing iodine (300mg/mL) was injected at 2-2.5 mL/kg body weight. Median scan delays from injection of contrast to starts of pancreatic parenchymal and portal venous phases were 40-50sec and 65-70sec, respectively. In most patients, the estimated transection line was at superior mesenteric vein, with modifications as needed for individual tumor locations and projected safety margin restrictions.

**Figure 2:**
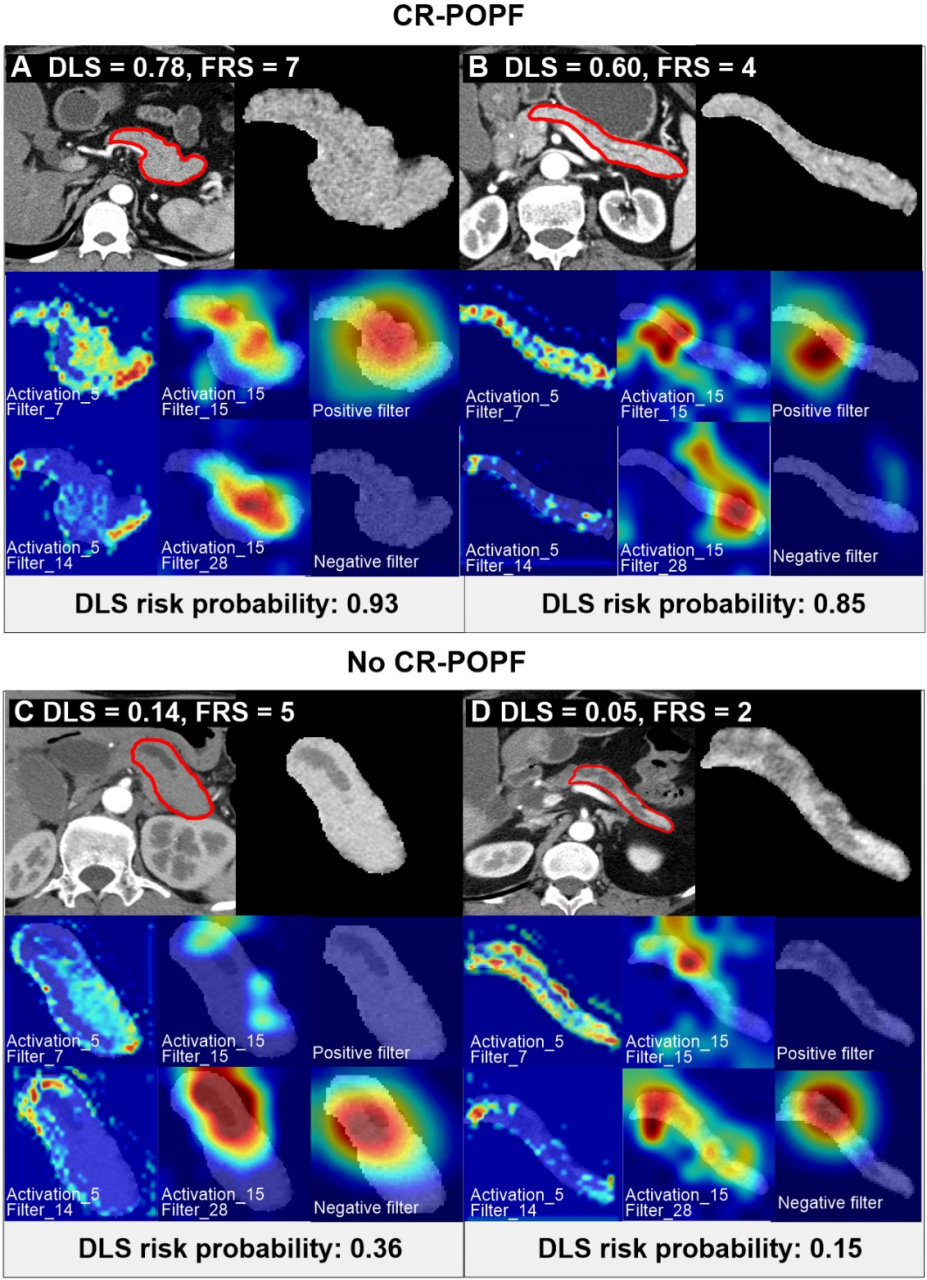
** Preoperative CT scan of patients with varying CR-POPF risks.** Two patients (A, B) ultimately developed CR-POPFs. FRS was 7 in patient A, and DLS was 0.78, both indicating high risk of CR-POPF; whereas a DLS of 0.60 in patient B suggested high probability of CR-POPF (0.85), despite intermediate FRS risk (FRS=4). The other two patients C and D did not develop CR-POPFs. FRS was 2 in patient C, and DLS was 0.05, both conferring low risk of CR-POPF; whereas a DLS of 0.14 in patient D showed low probability of CR-POPF (0.36) at intermediate FRS risk (FRS = 5). The first panel of each subgroup shows CT images and ROI regions used for DLS acquisition. For the second and third rows in each panel, the first and second columns shows the visualization of the activation layers of the ResCNN model, which assess the feature extraction (pancreatic parenchymal region and stump area), and the third column is the localization map that shows the important hot spots contributing to DLS for predicting non-CR-POPF/CR-POPF. The CT images were overlapped to reveal response locations. Corresponding histologic views of pancreatic stumps (patients B and D) are shown in **Supplemental Figure S4**.

Volumetric regions of interest (ROIs) in CT images (**Figure 2**) were segmented separately by four experienced abdominal radiologists (all with over 10 years of experience in pancreatic imaging), using open-source software (3D Slicer version 4.10; www.slicer.org). Segmentations were undertaken in pancreatic phase of transverse sections. In addition, pancreatic thickness, width, and remnant pancreatic volume were measured as previously published fixed CT classifiers (**Supplemental material S1**).

### Deep-learning (DL) model

The pipeline for the CR-POPF deep-learning model is depicted in **Supplemental Figure S1.** 2D-ROIs were input to the deep-learning model and DLS values were yielded as probabilities of CR-POPF. To further ensure a robust predictive exercise, the DL model received all pancreatic slices in each patient, conveying the average probability of CR-POPF. Further deep-learning training details can be found in **Supplemental material S3**. Intermediate activation layers were visualized to assess how the network carries the information from input to output to understand the feature extraction. The Gradient-weighted Class Activation Mapping (Grad-CAM) was used to produce a coarse localization map highlighting the important regions in the image for predicting the target concept (CR-POPF or non-CR-POPF). And the reconstructed localization maps were named as positive and negative filters later, which were also used to evaluate the class discrimination 36. Both Keras toolkit and Python 3.5 were needed to implement this model.

### Statistical analysis

Candidate risk factors included radiological DLS, clinical FRS, demographic parameters (sex, BMI, *etc*.), anastomotic technique (PD or PJ), surgeons and hospital sites, and fistula mitigation strategies (*e.g.* prophylactic octreotide or transanastomotic stent usage). Risk factors for CR-POPF demonstrating significance (*P <* 0.05) in univariate logistic regression analysis were then applied to multivariate logistic regression modeling. Strengths of associations were presented as odds ratios (ORs), with 95% confidence intervals (CIs). We also explored the potential for the radiologic DLS to enhance the clinical FRS in predicting CR-POPFs, especially at intermediate levels of FRS risk, pursuing logistic regression analysis in the predictive models. Their comparative performances were plotted as areas under the receiver operating characteristics (ROC) curve (AUC) by Delong method^37^. Diagnostic indices, such as accuracy, sensitivity, and specificity, were obtained with ROC-derived cut-off and compared using McNemar's test. To investigate a potential relation between DLS and histologic or morphologic changes, stepwise multiple linear regression tests were conducted (detailed in **Supplemental materials S4**).

All computations relied on standard software applications, including R v3.5.0 (R Project for Statistical Computing, Vienna, Austria), SPSS v25 (IBM Corp, Armonk, NY, USA), and MATLAB R2019a (MathWorks, Natick, MA, USA). Statistical significance was set at *P*<0.05.

### Usability testing

Double-blind usability testing addressed five functional aspects: predictive ability (*e.g.*, AUC, accuracy, *etc*.), learnability, efficiency, satisfaction, and memorability. Following training, an open-source DLS model was released online (https://github.com/lungproject/Pancreas), offering dual tutor-assisted sample cases. The external test cohort was evaluated independently and the DLS values were calculated as directed by the tutor on their own computers.

## Results

### Patient characteristics

Comparisons of clinical characteristics, intraoperative data, and postoperative histology in patients with and without CR-POPFs are presented in **Supplemental Table S4**. CR-POPFs developed in 95 of 583 patients (16.3%), with training, validation and test datasets marked by similar outcomes (*P* = 0.56). Median FRS values in the presence (*vs.* absence) CR-POPFs were significantly higher for training (6 [IQR: 4.5-7] *vs.* 3 [IQR: 2-5]), validation cohort (5.5 [IQR: 4.5-7] *vs.* 3 [IQR: 2-5]) and test cohort (7 [IQR: 5-7] *vs.* 4 [IQR: 2-6]). The same was true of median DLS values in training (0.54 [IQR: 0.46-0.65] *vs.* 0.38 [IQR: 0.29-0.45]), validation (0.54 [IQR: 0.46-0.60] *vs.* 0.38 [IQR: 0.27-0.48) and test cohort (0.57 [IQR: 0.55-0.67] *vs.* 0.37 [IQR: 0.28-0.44) subjects (all *P <* 0.001) (**Supplemental Figure S2**).

Interrater reproducibility for volumetric segmentation agreement was expressed as dice similarity coefficient and hausdorff distance in **Supplemental material S5**, and clinical outcomes are detailed in **Supplemental material S6**.

### Performance of DLS in predicting CR-POPF

**Figure 3:**
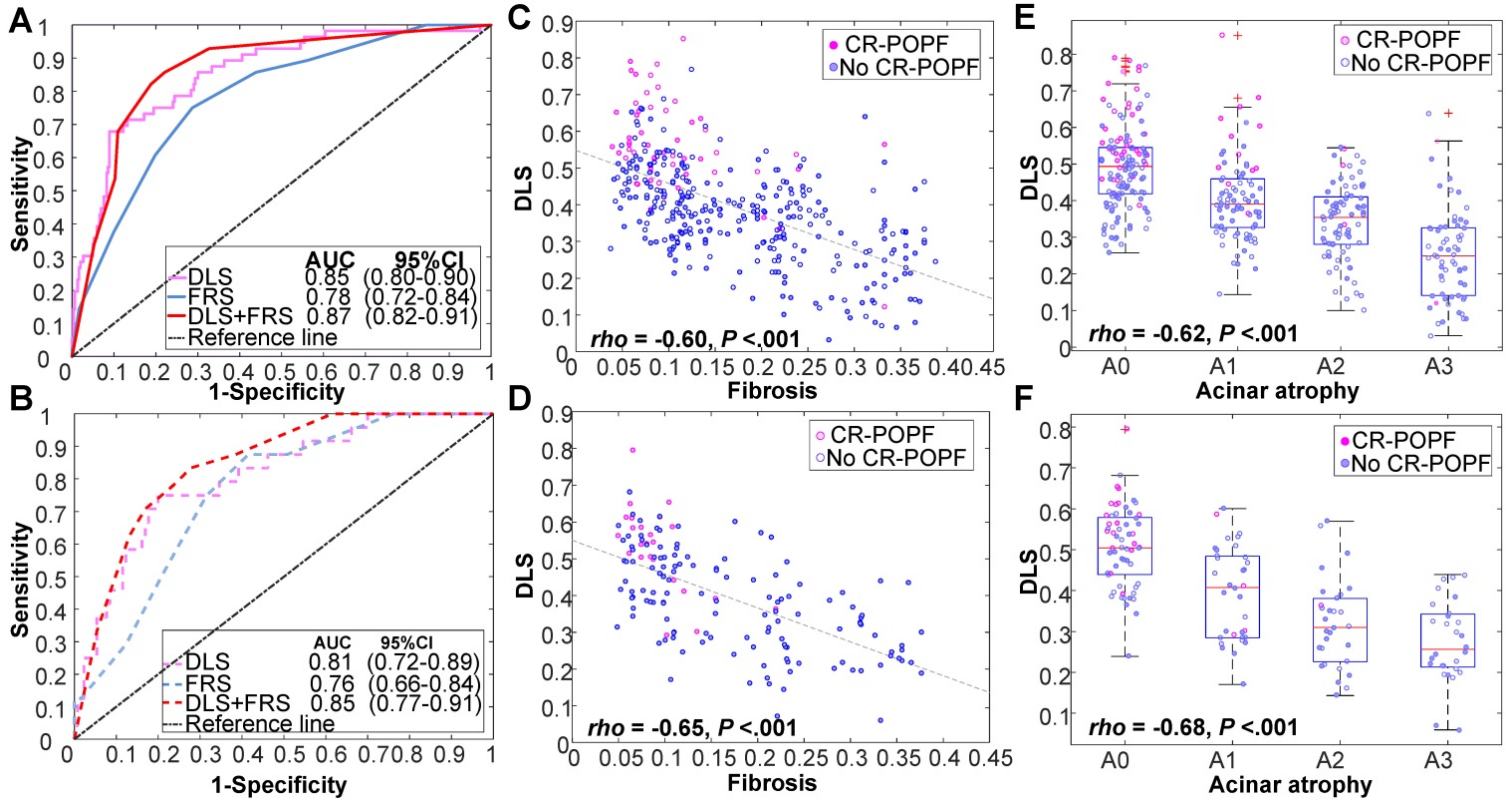
** Performance of DLS in various cohorts.** Receiver operating characteristic (ROC) curves of DLS, FRS, and DLS+FRS prediction models are plotted for the training cohort (A) and validation cohort (B). DLS is also shown to be significantly correlated with (C, D) fibrosis and (E, F) acinar atrophy in these two cohorts.

**Figure 2** showed CT images of four representative patients with different DLS and FRS. Through multivariate logistic regression analysis, DLS > 0.5 (OR = 12.23, 95% CI, 5.33-8.10; *P* < 0.001), incremental FRS increases (per point, OR = 1.43, 95% CI, 1.17-1.75; *P* < 0.001), octreotide use (OR = 3.70, 95% CI, 1.71-8.04; *P* = 0.001), and weight loss (OR = 0.34, 95% CI, 0.16-0.74; *P* = 0.006) proved to be independently associated with occurrences of CR-POPF (**Supplemental Table S5**), and DLS+FRS were generated consequently. DLS (cutoff: 0.5), FRS (cutoff: 5), and DLS+FRS (cutoff: 0.56) models were compared in differentiating CR-POPF presence or absence (**Table 1**). In terms of AUC, DLS (training: 0.85, 95% CI, 0.80-0.90, validation: 0.81, 95% CI, 0.72-0.89, test: 0.89, 95% CI, 0.79-0.96) significantly outperformed the FRS (training: 0.78, 95% CI, 0.72-0.84, *P* = 0.03, validation: 0.76, 95% CI, 0.66-0.84, *P* = 0.05, test: 0.73, 95% CI, 0.61-0.83, *P* < 0.001) (**Figure 3A, B**) in all three cohorts. Additionally, DLS proved higher predictive accuracy (training: 81.3% *vs.* 71.9%, *P <* 0.001; validation: 76.6% *vs.* 69.5%, *P* = 0.025) and specificity (training: 83.2% *vs.* 71.3%, *P <* 0.001; validation: 76.9% *vs.* 68.5%, *P* = 0.015) than did FRS, without significant compromise in sensitivity (training: 71.4% *vs.* 75.0%, *P* = 0.83; validation: 75.0% *vs.* 75.0%, *P* = 1.0) in the training and validation cohorts. In test cohort, the accuracy (*P* < 0.001), specificity (*P* = 0.008) and sensitivity (*P <* 0.001) of DLS were all significantly higher than those of FRS. Though the incorporation of FRS into DLS achieved higher AUCs of 0.87 (95% CI, 0.82-0.91), 0.85 (95% CI, 0.77-0.91) and 0.90 (95% CI, 0.80-0.96) in the training, validation and test cohorts, respectively, these improvements were not significant compared to DLS alone (training: *P* = 0.11, validation: *P* = 0.22, test: *P* = 0.52).

Previously published fixed CT classifiers (remnant pancreatic volume, MPD size, and pancreatic width or thickness) failed to surpass the DLS, and no further incremental gains in discriminatory capacity were achieved by adding these to the DLS (**Supplemental Figure S3**).

### FRS risk stratification analysis

Clinical FRS values served to stratify the train cohort by FRS risk level as low (38.7%), intermediate (47.1%) and high (14.2%). In 139 patients of low FRS risk, actual occurrences of CR-POPF accounted for only 5% of patients (n = 7). The accuracies of both DLS (96.4%; n = 134) and FRS (95.0%; n = 132) (*P* = 0.90) approached 100%. In 51 patients of high-FRS risk, actual CR-POPFs developed in 41.2% (n = 21). The DLS fared slightly (but not significantly) better than the FRS (60.7% *vs.* 41.2%; *P* = 0.053) in diagnostic accuracy. Confusion matrices of low- and high-risk patients in all cohorts are shown in **Supplemental Tables S5**.

**Figure 4:**
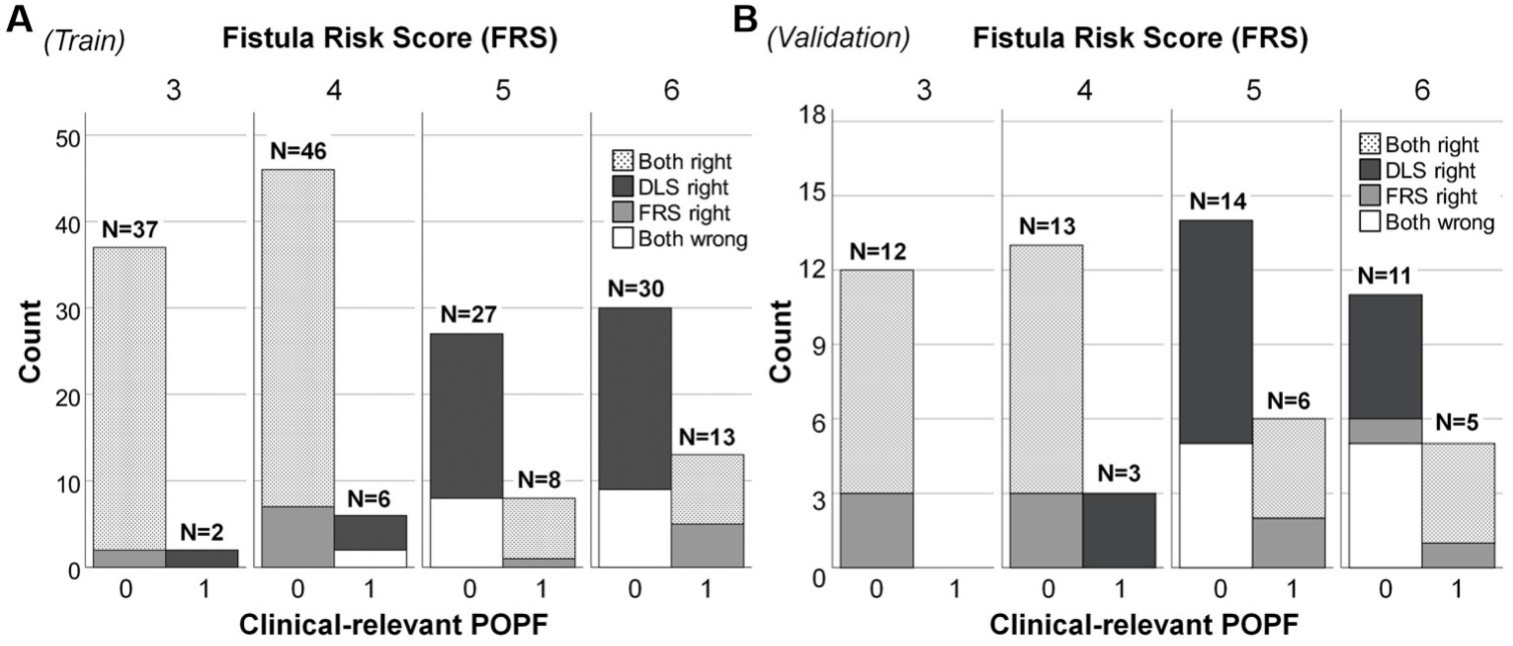
** Plotting of clinically relevant postoperative pancreatic fistula (CR-POPF) relative to Fistula Risk Score (FRS) and deep learning score (DLS) values in patients at intermediate risk.** In (A) the training cohort (n = 169) and (B) validation cohort (n = 64), totals above each column reflect occurrences according to risk score. Distributions of actual CR-POPFs are further stratified by FRS and DLS, shown as black (only DLS correct), white (DLS and FRS incorrect), gray (only FRS correct), and dotted (DLS and FRS correct) areas. Both DLS and FRS predicted more cases correctly at FRS values of 3 and 4 than at values of 5 and 6 (larger white areas). The DLS-only correctly classified cases (black areas) distributed mainly in the patients without CR-POPF, which was more than the FRS-only correctly classified cases (gray areas). Note. Intermediate risk: FRS is within the range of 3-6 points. 0: No CR-POPF; 1: CR-POPF.

In patients of intermediate FRS risk, the diagnostic accuracy of FRS was low in training cohort (61.5%), validation (56.3%) and test (65.8%) cohorts, whereas the DLS performed significantly better (training, 79.9%, *P* = 0.005; validation, 70.3%, *P* = 0.04; test, 92.1%, *P* = 0.013). In specific, the clinical FRS resulted in greater misclassification of training (57/140), validation (25/50) and test (11/33) patients as false positives, compared with the DLS (training: 26/140; validation: 16/50; test: 3/33), conferring specificities of 59.3% *vs.* 81.4% in the training cohort (*P* < 0.001), 50.0% *vs.* 68.0% in the validation cohort (*P* = 0.015), and 66.7 % *vs.* 90.9 % in the test cohort (*P* = 0.038), as detailed in **Figure 4** for each FRS score. A more specific confusion matrix is included as **Supplemental Table S6**; and details of AUC, specificity, and sensitivity are provided in **Supplemental Table S7**.

### Biology underpinnings of DLS

Histologically, the DLS significantly correlated with less fibrosis (training: *ρ* = -0.60; validation: *ρ* = -0.65; *P <* 0.01) and acinar atrophy (training: *ρ* = -0.62; validation: *ρ* = -0.68; *P <* 0.01), as indicated in **Figure 3C-F** DLS positively correlated with FRS and pancreatic parameters (volume, width, thickness) but negatively correlated with MPD diameter and gland texture (**Supplemental Table S8**). DLS also corresponded well with the hot-spotted pancreatic parenchyma and stump areas, representing the most important regions contributing to DLS (**Figure 2**).

Multivariate linear regression analysis (adjusted *r*^2^ = 0.51; *F* = 47.8; *P* < 0.001) further revealed that the DLS was independently associated with pancreatic fibrosis (coefficient = -0.17; *P* = 0.029), MPD (coefficient = -0.45;* P* < 0.001), and remnant pancreatic volume (coefficient = 0.14;* P* = 0.012), all contributing to 51.2% of DLS variability (**Supplemental Table S9**).

### Usability testing

In the test cohort, the user satisfaction score was 4 when using a 5-point scale. The external test cohort was prepared within 10 minutes, and the cases were run and DLS values listed within 1~2 minutes (see **Supplemental material S7** for usability testing results).

## Discussion

Recently, deep learning has become a popular tool for imaging analysis, fueled by its hierarchal automated learning capacities and the optimal parametric sets delivered. In utilizing the hidden layers of deep learning, the DLS described herein may well serve as a new, highly accessible and approachable means of routine patient examination. Within 1~2 minutes, DLS can be automatically generated from preoperative CT scans with excellent performance (AUC = 0.90 in external cohort), the latter being mandatory for nearly all patients in this setting.

Other traditional methods of quantitative imaging reported to date are burdened by complicated models and tedious manual measurements, whereas the DLS consolidates all local risk factors into one simplified model. We directly compared the DLS with almost all CT classifiers 5, 20-22, 38-41 cited in literature (remnant pancreatic volume, MPD diameter, and pancreatic width or thickness), discounting the common belief that deep learning always prevails. However, none of the above showed any superiority to DLS or any incremental benefit in combination with DLS, perhaps reflecting their innate correlations with DLS. One recent study 42 has claimed good results in 80 training (AUC = 0.82) and 37 test (AUC = 0.76) subjects by harnessing conventional radiomics analysis. Similarly, Kambakamba et al utilized machine learning-based texture analysis, which could achieve an impressive AUC of 0.95 in predicting POPF on 110 patients from a single institution 43. However, the present effort is the first to explore a deep-learning based method with significantly larger cohorts from different institutions, which is simpler to use and easier to interpret using one single biomarker (DLS, or the probability). Compared to classical textural analysis, the visualization of the developed deep learning model could highlight the important regions in the image, such as the pancreatic parenchyma and stump areas, for predicting CR-POPF or non-CR-POPF, which is helpful in revealing the biology underpinnings of the DLS. Additionally, our study further emphasized the super prediction ability in the sub-cohort with FRS-based intermediate risk, which is a grey zone warranting more objective and reliable predictors.

The radiologic DLS was designed to add more objective local features to the FRS and fully utilize preoperative CT studies, beyond mere recognition of MPD dilatation. Moreover, pancreatic texture (another determinant of FRS) is indirectly ascertained via DLS, perhaps enabling a strong sense of visually glandular fibrosis or atrophy on CT images. Our analysis also indicated that DLS may outperform the FRS at certain FRS risk levels. In low-risk subjects, in whom CR-POPFs are unlikely, FRS performance was excellent; whereas FRS values >6 clearly equated with high risk. Unfortunately, ~50% of cases gravitate to intermediate FRS risk, according to our data and that of others. CR-COPF rates at this intermediary level (~15**%**) and overall (~16**%**) are thus indistinguishable, hindering POPF prevention decisions. Contrastingly, the DLS significantly outperforms the FRS in excluding those patients who lack CR-POPFs, significantly improving specificity in the intermediate-risk group without altering sensitivity. Hence, we see no need for DLS calculation at very low FRS values, except if used as an alternative preoperative biomarker. The DLS is otherwise highly recommended at intermediate FRS levels and may help exclude falsely high risk assessments at FRS values > 6. In short, the DLS is intended for preoperative evaluation of CR-POPF risk in any manner deemed surgically appropriate. As in prior studies, octreotide use for mitigation and nutritional status (weight loss or BMI status) were also associated herein with CR-POPF risk. A comprehensive analysis of all parameters is likely to benefit in predicting postoperative CR-POPF.

Visualization of the convolution filters helped us demystify what has been learned in devising the DLS. Hot-spotted regions, such as remnant pancreatic volume and stump areas (Figure 2) were important contributors to the final concept, consistent with traditional CT 5 or MR 44 indices of these morphologic features. At a macroscopic level, DLS (Table S8) was significantly associated with MPD diameter, remnant pancreatic volume, pancreatic thickness or width, and softer gland texture, while showing a negative relation with pancreatic fibrosis and acinar atrophy in histologic preparations. Higher DLS thus corresponds with soft, non-fibrotic, large-sized pancreatic remnants and a non-distended MPD, signifying full tissue viability and fluid productivity and presenting greater challenge in suturing. Conversely, lower DLS attests to a hardened, fibrotic, and atrophic pancreas, with a dilated MPD and limited secretory capacity for easier anastomosis and lower risk of CR-POPF.

We do acknowledge some limitations of this study. The first issue is that our data accrued from four separate and independent centers attended by various research associates. Although different CT modalities were used, the scanners were standardized to 64-slice capability or better, with slice thickness <3 mm. The Hounsfield scale, an international standardization, was also upheld in all CT systems to control inner variabilities of different scanners. The FRS, however, relies on subjective evaluations of individual surgeons, creating opportunities for data inconsistencies. Furthermore, DLS and FRS values were generated exclusively at high-volume institutions and may not carry their predictive weight in lower-volume facilities equipped with earlier generations of scanners or staffed by fewer surgeons.

## Conclusion

In conclusion, quantitative preoperative CT assessment using AI is strongly predictive of CR-POPF in patients with pancreaticoenteric anastomoses following PD. The automated scores could reflect histomorphologic features pertaining to pancreatic duct, remnant pancreatic tissue volume and parenchymal fibrosis. The DLS is particularly helpful for patients with intermediate FRS risks in gauging the potential for CR-POPF. Future efforts would focus on its integration into image archiving and communication systems used for radiologic reporting or incorporation into surgical planning software to accommodate the preferences of surgeons, thus optimizing preoperative strategies, intraoperative decision-making, and even postoperative care.

## Supplementary Material

Supplementary figures and tables.

## Figures and Tables

**Table 1:** Performances of various predictive models in training, validation and test cohorts

	AUC (95% CI)	Accuracy (95% CI)	Sensitivity (95% CI)	Specificity (95% CI)
**Deep-learning score (DLS)**				
Training	0.85 (0.80, 0.90)	81.3 (77.2, 85.2)	71.4 (58.9, 82.1)	83.2 (78.9, 87.5)
Validation	0.81 (0.72, 0.89)	76.6 (70.1, 83.1)	75.0 (58.3, 91.7)	76.9 (70.0, 83.9)
Test	0.89 (0.79, 0.96)	87.1 (86.8, 87.5)	86.7 (59.5, 98.3)	87.3 (75.5, 94.7)
**Fistula Risk Score (FRS)**				
Training	0.78 (0.72, 0.84)	71.9 (67.4, 76.3)	75.0 (64.3, 85.7)	71.3 (66.3, 76.2)
Validation	0.76 (0.66, 0.84)	69.5 (62.3, 76.6)	75.0 (58.3, 91.7)	68.5 (60.8, 76.2)
Test	0.73 (0.61, 0.83)	72.9 (72.3, 73.4)	73.3 (44.9, 92.2)	72.7 (59.0, 83.9)
**DLS+FRS**				
Training	0.87 (0.82, 0.91)	81.3 (77.4, 85.5)	82.1 (71.4, 91.1)	81.2 (76.9, 85.8)
Validation	0.85 (0.77, 0.91)	75.9 (68.8, 83.1)	79.2 (62.5, 91.7)	75.4 (66.9, 83.1)
Test	0.90 (0.80, 0.96)	90.0 (89.7, 90.3)	86.7 (59.5, 98.3)	90.9 (80.0, 97.0)

AUC, area under receiver operating characteristic (ROC) curve; ACC, accuracy; CI, confidence interval.

## Abbreviations

CR-POPF: clinically relevant postoperative pancreatic fistula
CE-CT: contrast-enhanced computed tomography
PD: pancreatoduodenectomy
PJ: pancreaticojejunostomy
MPD: main pancreatic duct, FRS, Fistula Risk Score
DLS: deep-learning score
AUC: areas under the receiver operating characteristics curve
CI: confidence intervals
IQR: interquartile range
OR: odds ratio
