# Supplementary material for: Prediction of clinically relevant Pancreatico-enteric Anastomotic Fistulas after Pancreatoduodenectomy using deep learning of Preoperative Computed Tomography
Source: Theranostics. 2020 Aug 1;10(21):9779–88. doi: 10.7150/thno.49671 (PMC7449906; doi:10.7150/thno.49671)
Supplement: Supplementary file 1 — Supplementary figures and tables. [file thnov10p9779s1.pdf]

List of Supplemental Digital Content:

Supplemental material S1: Details of clinical data collection, FRS Calculation and Histology of pancreatic stump

Supplemental material S2: Manual morphologic measurements by CT

Supplemental material S3: Construction and training of deep-learning model

Supplemental material S4: Details of Statistical analysis

Supplemental material S5: Reproducibility of deep-learning model

Supplemental material S6: Detailed clinical outcomes of CR-POPF

Supplemental material S7: Usability testing in external validation cohorts

Table S1. Definition and grading of postoperative pancreatic fistula (POPF)

Table S2. Acquisition parameters for CT imaging in each cohort

Table S3. Fistula Risk Score (FRS) for predicting clinically relevant postoperative pancreatic fistula (CR-POPF) after pancreatoduodenectomy (PD)

Table S4. Univariate and multivariate logistic regression analyses of risk factors for clinically relevant postoperative pancreatic fistula (CR-POPF)

Table S5. Confusion matrix of outcomes using deep-learning-based score (DLS) or Fistula Risk Score (FRS) to predict clinically relevant postoperative pancreatic fistulas (CR-POPFs) in patients of intermediate (A), low (B) low and (C) high FRS risk

Table S6. Predictive performance of various methods at intermediate risk levels in training and validation cohorts

Table S7. Correlation of various factors with Deep Learning Signature (DLS) in predicting clinically significant postoperative pancreatic fistula (CR-POPF)

Table S8. Multivariate linear regression analysis of DLS

Figure S1: Schematic of ResCNN model

Figure S2: Distribution of FRS and DLS values in training and validation cohorts

Figure S3: Receiver operating characteristic (ROC) analysis of different models in predicting clinically relevant postoperative pancreatic fistula (CR-POPF)

Figure S4: Histology of pancreatic remnants in patient B (A, B) and D (C, D), corresponding with patients in Figure 2.

Supplemental Methods

Supplemental material S1: Details of clinical data collection, FRS Calculation and Histology of pancreatic stump

Pancreatic texture, subjectively gauged as soft or firm, was appraised by experienced lead surgeons through intraoperative palpation, regardless of histopathology. MPDs (mm) of pancreatic remnants were also measured intraoperatively by placing flexible rulers against cut surfaces of transected pancreas, or on the images of the most recent preoperative CT scans.

Fluid volumes and serum amylase levels were measured on postoperative Days 1, 3, 5, and 7. Two laminar drains were routinely placed ventral and dorsal to proximal ends of pancreatic anastomoses, and in 52 patients, an extra drain was positioned in the retroperitoneal space at end of surgery. Drains were regularly removed on Days 3-5 if drainage was not indicative of POPF. Otherwise, they remained in until POPFs resolved. A diagnosis of POPF was warranted if amylase levels of drainage fluid on or after postoperative Day 3 exceeded 3 times the upper limit in normal serum, according to guidelines of the International Study Group of Pancreatic Fistula (Table S3).

Specimens of the pancreatic stump were either retrieved from the local biobank or collected intraoperatively. All routinely processed samples were sectioned for hematoxylin and eosin (HE), Masson's trichrome, and Sirius Red staining to quantify fibrous tissue. Exocrine glandular atrophy (A) was graded according to extent of viability as A0 (75-100%), A1 (50-75%), A2 (25-50%) or A3 (0-25%) [S1]. Degrees of lipomatosis (L) were similarly graded as L0 (0-10%), L1 (10-20%), L2 (20-30%), or L3 (>30%) [S2,S3]. Histologic changes were evaluated by consensus of two dedicated pathologists (each with >10 years of experience in pancreatic pathology) who were blinded to clinical data and radiologic findings in each cohort.

S1. Hatano M, Watanabe J, Kushihata F, et al. Quantification of pancreatic stiffness on intraoperative ultrasound elastography and evaluation of its relationship with postoperative pancreatic fistula. International surgery 2015; 100(3):497-502.

S2. Watanabe H, Kanematsu M, Tanaka K, et al. Fibrosis and Postoperative Fistula of the Pancreas: Correlation with MR Imaging Findings—Preliminary Results. Radiology 2013; 270(3):791-799.

S3. Gaujoux S, Cortes A, Couvelard A, et al. Fatty pancreas and increased body mass index are risk factors of pancreatic fistula after pancreaticoduodenectomy. Surgery 2010; 148(1):15-23.

Supplemental material S2: Manual morphologic measurements by CT

Two radiologists (both blinded to postoperative outcomes) manually measured maximum pancreatic thickness (anterioposterior diameter in transverse sections) and width (caudocephalic diameter in coronal sections) at estimated transection line and remnant pancreatic volume (RPV) during pancreatic parenchymal phase. Pancreatic thickness and MPD diameter were also measured in axial sections. The full width of pancreas was determined at estimated transection line in coronal sections. RPV was calculated by subtracting remnant MPD volume (if MPD diameter ≥3 mm) from overall pancreatic remnant volume (size of segmentation set by 3D Slicer or other segmentation software).

Supplemental material S3: Construction and training of deep-learning model

Structure of the deep learning model

The constructed model is similar to the Resnet18 but with fewer filters, and the architecture was shown in supplemental Figure S1. The architecture was comprised with one convblock (including a 3×3 convolutional layer followed by a batch normalization layer and a rectified linear unit (ReLU) activation layer), 8 residual blocks (Resblock), and one fully connected layer. Finally, a softmax activation layer was connected to the last fully connected layer, which was used to yield the prediction probabilities of nodule candidates. To prevent overfitting, one dropout layer with probability of 0.3 was added to the fully connected layers. Additionally, the model was optimized using the binary cross entropy loss function.

Preparation of the input images

Due to the low prevalence of CR-POPF (~15%), positive/negative case balance was achieved by selecting representative slices from each negative cases. The smallest square, which includes the whole segmentation results in each slice, was used as a ROI to input. There were 11053 ROIs were generated for training. Before inputting to the DL model, all the ROIs were resized to the

same size (64×64) using cubic spline interpolation, and were standardized by z-score normalization, which meant the tumor image was subtracted by the mean intensity value and divided by the standard deviation of the image intensity, to reduce the effect of different equipment and different reconstruction parameters.

Training of the deep learning model

During the training, binary cross entropy was employed as the loss function and the Adam optimizer with an initial learning rate = 0.0001, beta_1=0.9, beta_2=0.999 was used. The learning rate was reduced by a factor of 5 if no improvement of the loss of the validation dataset was seen for a 'patience' number (n=10) of epochs. The batch size was set to 64.

In order to reduce the risk of overfitting, several techniques were deployed. 1) Augmentation: During the training, augmentation including width/height-shift, horizontal/vertical-flip, rotation and zoom were used to expand the training dataset to improve the ability of the model to generalize. 2) Regularization: L2 regularization was used, which added a cost to the loss function of the network for large weights. As a result, a simpler model that was forced to learn only the relevant patterns in the training data would be obtained. 3) Dropout: Dropout layer, which would randomly set output features of a layer to zero during the training process, was added. 4) Early stop: During training, the model is evaluated on the validation dataset after each epoch. The training was stopped after waiting an additional 30 epochs since the validation loss started to degrade. 5) Cross-validation: The number of the filters, the learning rate, and batch size was determined with five-fold-cross validation under the patient level, and the combination that yielded the best average accuracy on the internal-validation folds was chosen.

Supplemental material S4: Details of Statistical analysis

Continuous variables, expressed as mean ± standard deviation (std) or median and interquartile range (IQR, 25th-75th percentile) accordingly, were compared via Kruskal-Wallis or Mann-Whitney U test with Bonferroni correction. Categorical variables were expressed as counts and percentages, using X2 or Fisher's exact test as warranted for comparisons.Interrater agreement of volumetric segmentations and DLS estimates was indicated by intraclass correlation

coefficients (ICCs) of the two radiologists. To assess multicollinearity, variance inflation factor (<5) and Spearman's correlation ($r<0.7$) were used.

SUPPLEMENTAL RESULTS

Supplemental material S5: Reproducibility of deep-learning model

Interrater agreement was expressed as Dice similarity coefficient (DSC), which measured volumetric overlap, and Hausdorff distance (HD), representing the maximum distance from one set to the nearest point in the other set. DLS agreement was excellent in the training and validation cohorts, with interrater ICCs of 0.96 (95% CI: 0.95-0.97), 0.92 (95% CI: 0.89-0.94) and 0.93 (95% CI: 0.91-0.96), respectively. Segmentation agreement was fair in the training and validation cohorts, with DSCs of 88.90±2.84%, 82.11±5.12% and 85.02±4.46%, HDs of 8.03±0.59 mm, 9.55±0.71 mm and 9.10±0.65 mm, respectively.

Supplemental material S6: Detailed clinical outcomes of CR-POPF

Antibiotics were administered for fistula management in 15.6% of patients (91/583); supplemental total parenteral nutrition (TPN) was initiated in 6% (35/583); and percutaneous drainage was used in 3.5% (18/583). There were 56 readmissions (9.6%) and 15 reoperations (2.5%). Patients with CR-POPFs experienced more non-fistulous complications (biochemical POPF, 26%; CR-POPF, 77%; $p<0.001$), more ICU confinement (biochemical POPF, 0%; CR-POPF, 24%; $p<0.001$), and progressively longer median hospital stays (biochemical POPF, 6 days; CR-POPF, 12 days, $p<0.001$). There was no 90-day mortality directly attributable to pancreatic fistulas in this series.

Supplemental material S7: Usability testing

Usability testing of the DLS model examined end-user perspectives (Nielsen's usability definition). The ease with which a user accessed the model constituted the main testing point. There were five aspects of testing: accuracy and error, learnability, efficiency, satisfaction, and memorability.

1. Accuracy and error:

In all cohorts, the detailed predictive ability was shown in the main text and tables. During testing, there was a tiny error in running test_allpatient.py on the computer in institution C. All DLS values were correctly outputted. In institution D, five first attempts failed since the ROIs were not prepared for these cases. All were corrected in second attempts.

2. Learnability:

Because there was no programming needed when running all the scripts, and the open-source applications were already installed by testers previously (eg, 3D Slicer for segmentation, Python for running of scripts), the lists of DLS were easily acquired. The open-source DLS model and instructions were released online (https://github.com/lungproject/Pancreas).

3. Efficiency:

All 70 cases in the test cohort were run and DLS values listed in 1~2 minutes.

4. Satisfaction:

Given acceptable accuracy and quick performance, both testers rated the model as 4 on a 5-point satisfaction scale (very satisfied=5, satisfied=4, neutral=3, dissatisfied=2, very dissatisfied=1).

Note: testers indicated in feedback that in addition to DLS output and Y/N prediction of CR-POPF, probabilities were also desired. Our model was then revised accordingly.

5. Memorability:

Function and output were simple, so testers encountered no problems.

Supplemental Tables

Table S1. Definition and grading of postoperative pancreatic fistula (POPF)

**POPF**	
POPF	The drain fluid has an amylase content greater than 3 times the upper limit of the normal serum value for the institution, measure on or after the 3rd postoperative day
Biochemical POPF (POPF A)	Transient, asymptomatic fistulas, with elevated drain amylase levels not requiring treatment or deviation in clinical management
POPF B	Symptomatic, clinically apparent fistulas that require diagnostic evaluation and therapeutic management using antibiotic therapy, octreotide infusion, supplemental nutrition (total parenteral nutrition [TPN]), transfusion, maintenance of drains for a prolonged period (>21 days), angiographic procedures for bleeding, additional percutaneous or endoscopic drainage, or any combination thereof
POPF C	Severe, clinically significant fistulas that require major deviations in clinical management; single or multiple organ failure, and or Reoperation, POPF-related Death

POPF indicates failure of healing/sealing at pancreaticoenteric anastomoses.

Note: these three grades of POPF severity were defined according to the International Study Group for Pancreatic Fistulas clinical criteria (2016).

Table S2. Fistula Risk Score (FRS) for predicting clinically relevant postoperative pancreatic fistula (CR-POPF) after pancreatoduodenectomy (PD)

Risk factor	Parameter	Points
Gland texture	Firm	0
	Soft	2
Pathology	Pancreatic adenocarcinoma or pancreatitis	0
	Other patholo	1
Pancreatic duct diameter	≥5 mm	0
	4 mm	1
	3 mm	2
	2 mm	3
	≤1 mm	4
Intraoperative blood loss	≤400 ml	0
	401-700 ml	1
	701-1000 ml	2
	>1000 ml	3
	Total 0-10points	

Table S3. Acquisition parameters for CT imaging in each cohort

Characteristic	Training (N=359)	Validation (N=154)	Test (N=70)	*p*
Manufacturer				.32
SIEMENS	76 (21.17)	39 (25.32)	42 (60.0)	
GE MEDICAL	45 (12.53)	26 (16.88)	0	
PHILIPS	94 (26.18)	35 (22.73)	28 (40.0)	
TOSHIBA	144 (40.11)	54 (35.06)	0	
Kilovoltage peak, kVp				-
120	120	120	120	
Current, mA				.12
Median (range)	288 (90-670)	304 (84-675)	331 (121-645)	
CT slice thickness, mm				.19
Median (range)	2 (1-3)	2 (1-3)	3 (1-3)	
CT pixel spacing, mm				.61
Median (range)	0.78 (0.52-0.98)	0.72(0.52-0.98)	0.68 (0.54-0.79)	
Scan acquisition time delay, sec				.93
Median (range)	47 (40-50)	47 (40-50)	46 (40-50)	

Data expressed as n (%) or median (range)

Table S4. Demographic and clinicopathologic characteristics of patients

Characteristics	Training cohort			Validation cohort			Test cohort		
	No POPF (N=303)	POPF (N=56)	*P*	No POPF (N=130)	POPF (N=24)	*P*	No POPF (n=55)	POPF (n=15)	*P*
**Patient characteristics**									
Age, mean (SD), yr	59.3 (9.8)	58.5 (10.2)	.49	60.4 (9.1)	58.5 (5.1)	.51	60 (9.4)	57 (8.0)	.12
BMI , No. (%)			**.045**			.06			.26
≥25 kg/m^2	55 (18.15)	17 (30.36)		25 (19.23)	9 (37.50)		8 (14.55)	4 (26.67)	
<25 kg/m^2	248 (81.85)	39 (69.64)		105 (80.77)	15 (62.50)		47 (85.45)	11 (73.33)	
Sex,No. (%)			.66			.66			.73
Male	178 (58.75)	31 (55.36)		68 (52.31)	14 (58.33)		21 (38.18)	5 (33.33)	
Female	125 (41.25)	25 (44.64)		62 (47.69)	10 (41.67)		34 (61.82)	10 (66.67)	
Dabetes mellitus, No. (%)			.73			.22			.65
Yes	74 (24.42)	12 (21.43)		36 (27.69)	10 (41.67)		18 (32.73)	4 (26.67)	
No	229 (75.58)	44 (78.57)		94 (72.31)	14 (58.33)		37 (67.27)	11 (73.33)	
Jaundice			.24			.38			.97
Yes	156 (51.49)	34 (60.71)		64 (49.23)	9 (37.50)		29 (52.73)	8 (53.33)	
No	147 (48.51)	22 (39.29)		66 (50.77)	15 (62.50)		26 (47.27)	7 (46.67)	
History of Smoking, No. (%)			.10			.37			.64
Yes	184 (60.73)	27 (48.21)		70 (53.85)	10 (41.67)		33 (60)	10 (66.67)	
Never	119 (39.27)	29 (51.79)		60 (46.15)	14 (58.33)		22 (40)	5 (33.33)	
Alcohol abuse, No. (%)			.55			.07			.97
Yes	109 (35.97)	23 (41.07)		55 (42.31)	5 (20.83)		18 (32.73)	5 (33.33)	
No	194 (64.03)	33 (58.93)		75 (57.69)	19 (79.17)		37 (67.27)	10 (66.67)	
Weight loss, No. (%)			**.008**			**.047**			**.004**
≥3 kg	156(51.49)	34 (60.71)		64 (49.23)	9 (37.50)		34 (61.82)	3 (20)	
<3 kg	147(48.51)	22 (39.29)		66 (50.77)	15 (62.50)		21 (38.18)	12 (80)	
**Morphologic measurements by CT**									
Volume, mean (SD), cm^3	22.5 (13.5)	37.0 (17.8)	**<.001**	24.1 (18.3)	45.6 (19.1)	**<.001**	17.0 (12.0)	43.9 (18.7)	**.001**
Thickness, mean (SD),mm	22.1 (7.1)	24.5 (7.6)	**.006**	22.1 (7.9)	28.1 (5.6)	**.001**	20.5 (5.6)	24.6 (6.4)	**.027**
Width, mean (SD),mm	19.9 (6.0)	23.4 (8.7)	**.036**	20.0 (7.6)	24.9 (6.8)	**.015**	18.7 (5.0)	24.25 (7.8)	.075
**Operative and intraoperative factors**									
Anastomosis, No. (%)			.76			.19			.93
A	110 (36.30)	23 (41.07)		45 (34.62)	7 (29.17)		30 (54.55)	8 (53.33)	
B	52 (17.16)	10 (17.86)		22 (16.92)	5 (20.83)		25 (45.45)	7 (46.67)	
Operative time, mean (SD), min	484 (73)	493.4 (59)	.15	489 (65)	115 (79)	.06	488 (74)	496 (58)	.55
Blood loss, mean (SD), ml	443.6 (134.5	513.1(190.7)	**.011**	436.2 (123.9)	549.0(226.3)	**.03**	469.6 (158.7)	528.4 (198.1)	.27
Reconstruction, No. (%)			.66			.83			.43

PJ	162 (53.47)	33 (58.93)		66 (50.77)	13 (54.17)		26 (47.27)	7 (46.67)	
PG	141 (46.53)	23 (41.07)		64 (49.23)	11 (45.83)		29 (52.73)	8 (53.33)	
**Surgeon's evaluation**									
Pancreatic texture, No. (%)			**<.001**			**<.001**			**.018**
soft	100 (33.00)	40 (71.43)		45 (34.62)	19 (79.17)		24 (43.64)	4 (26.67)	
hard	203 (67.00)	16 (28.57)		85 (65.38)	5 (20.83)		31 (56.36)	11 (73.33)	
MPD, mean (SD), mm	4.98 (2.93)	2.61 (1.62)	**<.001**	5.45 (3.77)	2.79 (1.15)	**.001**	4.70 (2.42)	1.60 (1.05)	**.001**
**Histopathology of pancreatic stump**									
Fibrosis percentage, mean(SD)	0.18 (0.09)	0.11 (0.06)	**<.001**	0.18 (0.10)	0.09 (0.04)	**<.001**	-	-	-
Acinar atrophy, No. (%)			**<.001**			**<.001**	-	-	-
A0	96 (31.68)	39 (69.64)		41 (31.54)	19 (79.17)				
A1	71 (23.43)	12 (21.43)		30 (23.08)	4 (16.67)				
A2	76 (25.08)	3 (5.36)		29 (22.31)	1 (4.17)				
A3	60 (19.8)	2 (3.57)		28 (21.54)	0 (0)				
Lipomatosis, No. (%)			.66			.06	-	-	-
L0	83 (27.39)	14 (25.00)		32 (24.62)	6 (25)				
L1	144 (47.52)	9 (16.07)		65 (50)	12 (50)				
L2	71 (23.43)	12 (21.43)		28 (21.54)	4 (16.67)				
L3	5 (1.65)	5 (8.93)		5 (3.85)	2 (8.33)				
Indications, No. (%)			**.013**			**.042**			**.003**
PDAC+CP	291 (96.04)	31 (55.36)		89 (68.46)	13 (54.17)		22 (40)	3 (20)	
Other	12 (3.96)	25 (44.64)		41 (31.54)	11 (45.83)		33 (60)	12 (80)	

Note. Data expressed as mean±SD, unless otherwise specified; *P* values in bold <0.05
BMI, body mass index; DM, diabetes mellitus; anastomosis A, end-to-side; anastomosis B, duct-to-mucosa;
PJ, pancreaticojejunostomy; PG, pancreaticogastrostomy; MPD, main pancreatic duct; PDAC, pancreatic ductal adenocarcinoma;
CP, chronic pancreatitis
History of weight loss implies ≥3-kg weight loss over previous 6 months.
Other indicates cystic neoplasms, ampullary cancer, neuroendocrine tumors, cholangiocarcinoma, duodenal carcinoma, intraductal papillary mucinous neoplasm, etc, aside from PDAC and CP.

Table S5. Univariate and multivariate logistic regression analyses of risk factors for clinically relevant postoperative pancreatic fistula (CR-POPF)

	**Univariate analysis**		**Multivariate analysis**	
	Odds Ratio (95% CI)	*P*	Odds Ratio (95% CI)	*P*
**Age**	0.99 (0.96-1.02)	.60	-	
**BMI (>25 kg/m^2)**	1.97 (1.04-3.73)	.039	0.721(0.324-1.605)	.423
**Sex (M)**	1.15 (0.65-2.01)	.64	-	
**Diabetes**	0.84 (0.42-1.68)	.63	-	
**Jaundice**	1.46 (0.81-2.61)	.21	-	
**Smoking status**	0.60 (0.34-1.07)	.083	-	
**Weight loss**	0.44 (0.24-0.80)	.007	0.34(0.16-0.74)	.006
**Alcohol abuse**	1.24 (0.69-2.22)	.47	-	
**Operative time**	1.00 (1.00-1.01)	.36	-	
**Stump mobilization**	0.99 (0.96-1.01)	.16	-	
**Anastomosis**	1.14 (0.63-2.07)	.66	-	
**PG (vs PJ)**	1.26 (0.70-2.26)	.445	-	
**Octreotide**	3.90 (2.13-7.12)	<.001	3.70 (1.71-8.04)	.001
**Stent**	1.47 (0.75-2.85)	.262	-	
**Volume (>22.27cm^3)**	4.01 (2.07-7.76)	<.001	1.24 (0.50-3.08)	.648
**Thickness (>2.26 cm)**	2.19 (1.21-4.00)	.010	0.59 (0.24-1.44)	.244
**Width (>2.04 cm)**	1.83 (1.02-3.29)	.044	1.12 (0.47- 2.65)	.801
**FRS (per point)**	1.70 (1.45-1.99)	<.001	1.43 (1.17-1.75)	<.001
**DLS (>0.5)**	15.1 (7.79-29.35)	<.001	12.23 (5.33-8.104)	<.001

Factors subjected to multivariable analysis were those showing significance at *P*<0.05 (bolded) in univariate analysis
Cutpoints for continous variables obtained by maximizing Youden's index (sensitivity+specificity-1) in individual receiver operating characteristics curve analysis
CI, confidence interval; BMI, body mass index; PJ, pancreaticojejunostomy; PG, pancreaticogastrostomy; FRS, fistula risk score; DLS, deep-learning signature

Table S6. Confusion matrix of outcomes using deep-learning-based score (DLS) or Fistula Risk Score (FRS) to predict clinically relevant postoperative pancreatic fistulas (CR-POPFs) in patients of intermediate (A), low (B) low and (C) high FRS risk

A: Confusion matrix of DLS (FRS) in patients of intermediate FRS risk (FRS: 3~6)

Training			Validation			Test		
	Actual			Actual			Actual	
	No	Yes		No	Yes		No	Yes
Predicted No	**114 (83)**	8 (8)	Predicted No	**34 (25)**	3 (3)	Predicted No	**30 (22)**	0 (2)
Predicted Yes	26 (57)	**21 (21)**	Predicted Yes	16 (25)	**11 (11)**	Predicted Yes	3 (11)	**5 (3)**
Total	140	29	Total	50	14	Total	33	5

B: Confusion matrix of DLS in patients of low FRS risk (FRS: 0~2)

Training			Validation			Test		
	Actual			Actual			Actual	
	No	Yes		No	Yes		No	Yes
Predicted No	**130**	3	Predicted No	**56**	3	Predicted No	**13**	1
Predicted Yes	2	**4**	Predicted Yes	8	0	Predicted Yes	0	**1**
Total	132	7	Total	64	3	Total	13	2

C: Confusion matrix of DLS in patients of high FRS risk (FRS: 7~10)

Training			Validation			Test		
	Actual			Actual			Actual	
	No	Yes		No	Yes		No	Yes
Predicted No	**16**	6	Predicted No	**3**	7	Predicted No	**5**	3
Predicted Yes	14	**15**	Predicted Yes	3	**10**	Predicted Yes	4	**5**
Total	30	21	Total	6	17	Total	9	8

The confusion matrix of outcomes by FRS was in parentheses.
Yes/No corresponds with presence/absence of clinically relevant postoperative pancreatic fistula.
The optimal cutpoint of DLS was 0.5 and the cutpoint of FRS was 5.
Cases in bold indicate those correctly predicted by DLS or FRS in training and 2 validation cohorts.

Table S7. Predictive performance of various methods at intermediate risk levels in training, validation and test cohorts

	AUC (95%CI)	Accuracy (95%CI)	Sensitivity (95%CI)	Specificity (95%CI)
**Deep-learning score (DLS)**				
Training	0.82 (0.74, 0.9)	79.9 (73.4,85.2)	72.4 (55.2, 89.7)	81.4 (75.0, 87.1)
Validation	0.75 (0.63, 0.85)	70.3 (58.6,81.3)	78.6 (57.1, 100.0)	68.0 (54.0, 80.0)
Test	0.96 (0.83, 0.99)	92.1 (91.7,92.5)	100.0 (47.8, 100.0)	90.9 (75.7, 98.1)
**Fistula Risk Score (FRS)**				
Training	0.69 (0.58, 0.78)	61.5 (54.4, 68.9)	72.4 (53.5, 89.7)	59.3 (51.4, 67.5)
Validation	0.67 (0.54, 0.81)	56.3 (45.3, 68.8)	78.6 (57.1,100.0)	50.0 (38.0, 64.0)
Test	0.68 (0.51, 0.82)	65.8 (64.6, 67.0)	60.0 (14.7,94.7)	66.7 (48.2, 82.0)
**DLS+FRS**				
Training	0.83 (0.77,0.89)	80.5 (73.9, 86.9)	72.4 (52.8, 87.3)	82.1 (74.8, 88.1)
Validation	0.77 (0.65,0.87)	71.8 (60.1, 82.8)	78.6 (49.2, 95.3)	70.0 (55.4 - 82.1)
Test	0.99 (0.88, 1.0)	97.4 (97.2, 97.5)	100.0 (47.8, 100.0)	96.9 (84.2, 99.9)

AUC, area under receiver operating characteristic (ROC) curve; CI, confidence interval

Table S8. Correlation of various factors with Deep Learning Signature (DLS) in predicting clinically significant postoperative pancreatic fistula (CR-POPF)

		Thickness	Width	Gland texture	Pathology	MPD	Blood loss	Pancreatic Volume	DLS	FRS
**Width**	Training	0.63**								
	Validation	0.66**								
**Gland texture**	Training	-0.31**	-0.28**							
	Validation	-0.37**	-0.37**							
**Pathology**	Training	0.008	0.12**	-0.008						
	Validation	-0.06	-0.11	0.071						
**MPD**	Training	-0.29**	-0.22**	0.48**	0.004					
	Validation	-0.42**	-0.41**	0.55**	0.083					
**Blood loss**	Training	0.061	0.079	-0.022	0.086	-0.046				
	Validation	0.18**	0.10	-0.27**	0.008	0.098				
**Pancreatic Volume**	Training	0.62**	0.62**	-0.42**	0.049	-0.49**	0.02			
	Validation	0.77**	0.65**	-0.27**	-0.027	-0.51	0.11			
**DLS**	Training	0.40**	0.31**	-0.51**	-0.025	-0.65**	0.019	0.53**		
	Validation	0.44**	0.38**	-0.48**	-0.092	-0.65**	0.086	0.52**		
**FRS**	Training	0.34**	0.30**	-0.76**	0.087	-0.86**	0.31**	0.51**	0.60**	
	Validation	0.46**	0.43**	-0.82**	0.034	-0.85**	0.49**	0.54**	0.60**	
**CR-POPF**	Training	0.16**	0.16**	-0.29**	-0.069	-0.36**	0.042	0.31**	0.44**	0.36**
	Validation	0.32**	0.21**	-0.33**	-0.046	-0.34**	0.014	0.36**	0.39**	0.34**

Note. Correlation is significant at the 0.01 level (2-tailed). Pathology: pancreatic duct adenocarcinoma or chronic pancreatitis; MPD: main pancreatic duct; DLS: deep-learning score; FRS: fistula risk score

Table S9. Multivariate linear regression analysis of DLS

Parameters	Standardized coefficients (β)	*P*	95.0% CI (β) Lower	Upper	R^2	VIF
**Fibrosis**	**-0.167**	**.029**	**-0.315**	**-0.018**	**-0.116**	**4.195**
Lipomatosis	-0.092	.210	-0.237	0.049	0.088	3.931
Atrophy	0.058	.124	-0.014	0.134	-0.069	1.045
**MPD**	**-0.445**	**<.001**	**-0.541**	**-0.346**	**-0.432**	**1.826**
**Volume**	**0.138**	**.012**	**0.033**	**0.245**	**0.169**	**2.164**
Texture	0.030	.558	-0.071	0.130	0.031	1.918
Width	0.007	.890	-0.098	0.111	0.007	2.099
Thickness	0.036	.480	-0.064	0.135	0.038	1.882

Parameters in bold showed significance in multivariate linear regression analysis
All VIF values <5 indicate no collinearity among parameters
MPD, main pancreatic duct; CI, confidence interval; R2, partial correlation coefficient; VIF, variance inflation factor.

Supplementary Figures

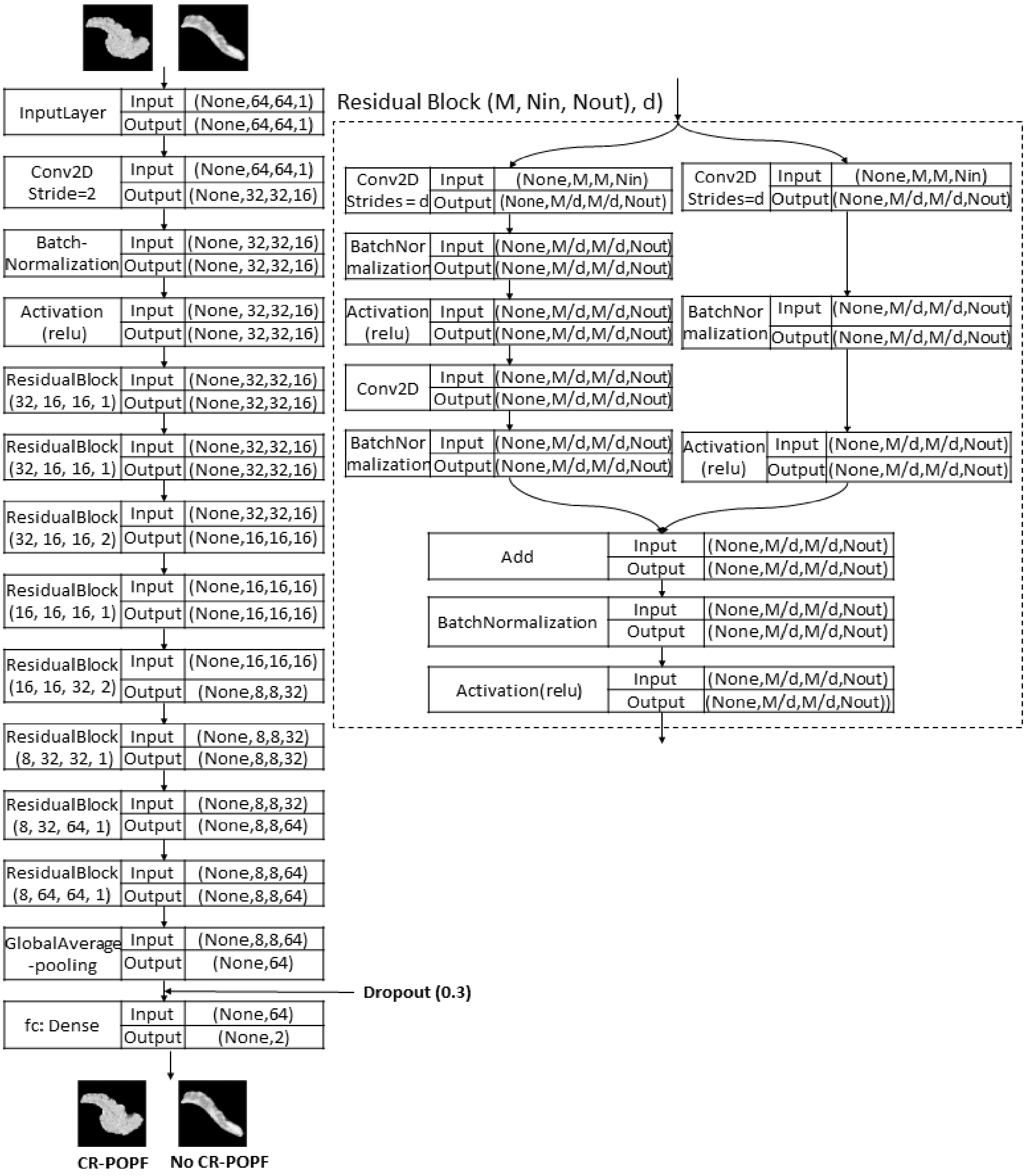

Figure S1. Schematic of ResCNN model (convolutional layers of 3x3 kernel size, batch normalization, pooling, and drop-out layers).

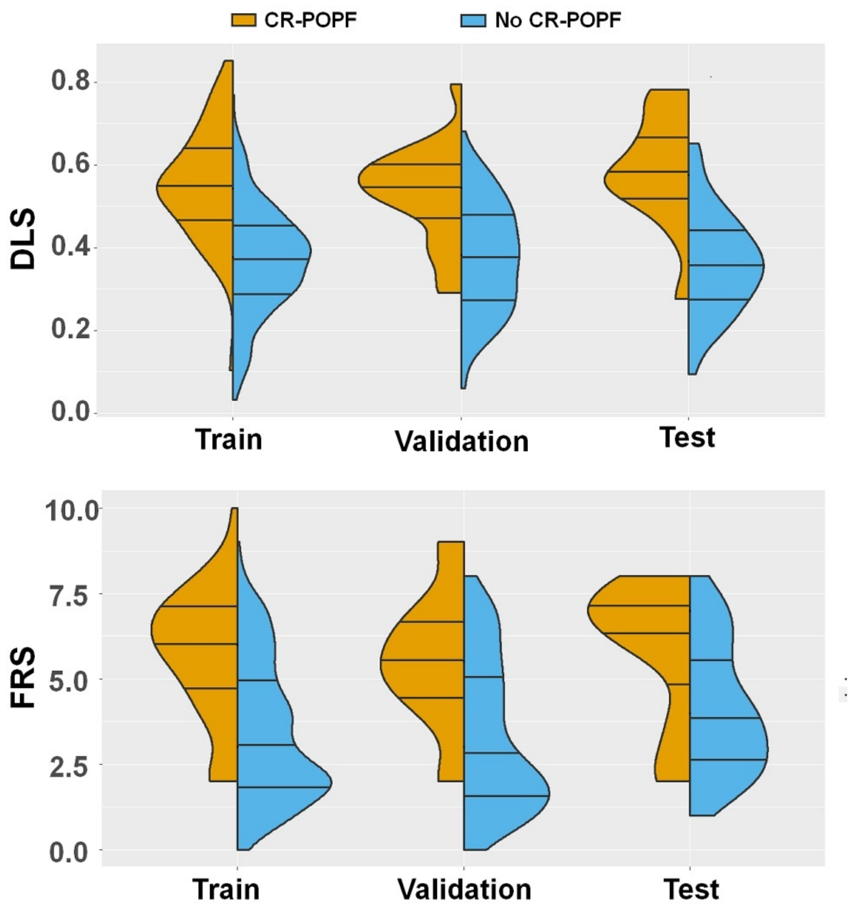

Figure S2. Distribution of FRS and DLS values in training and validation cohorts

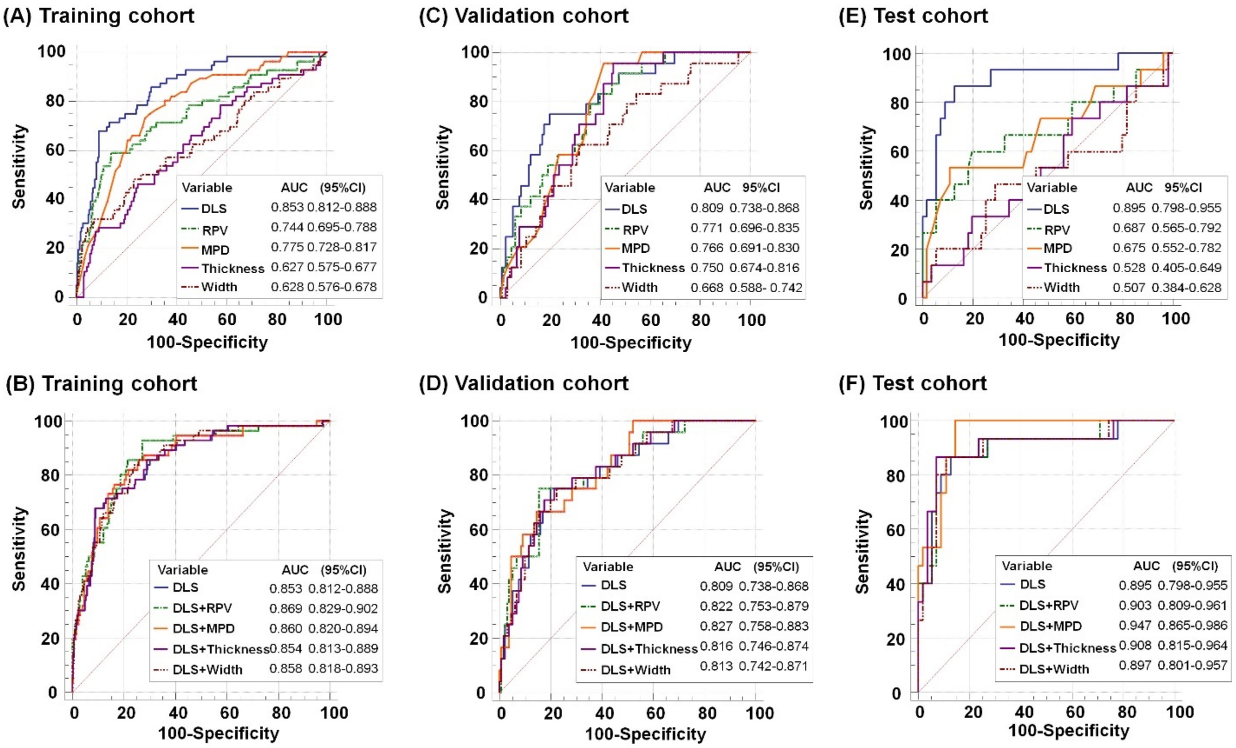

Figure S3. Receiver operating characteristic (ROC) analysis of different models in predicting clinically relevant postoperative pancreatic fistula (CR-POPF). A, C, and E are the comparison of DLS, remnant pancreatic volume (RPV), main pancreatic duct (MPD), pancreatic thickness and width in the training, validation, and test cohort, respectively. The area under the ROC curve (AUC) was highest for DLS, surpassing all other single predictors in all three cohorts. B, D, and F show the comparison of DLS with RPV, MPD, thickness, and width added, which showed the addition of these predictors conferred no incremental improvement.

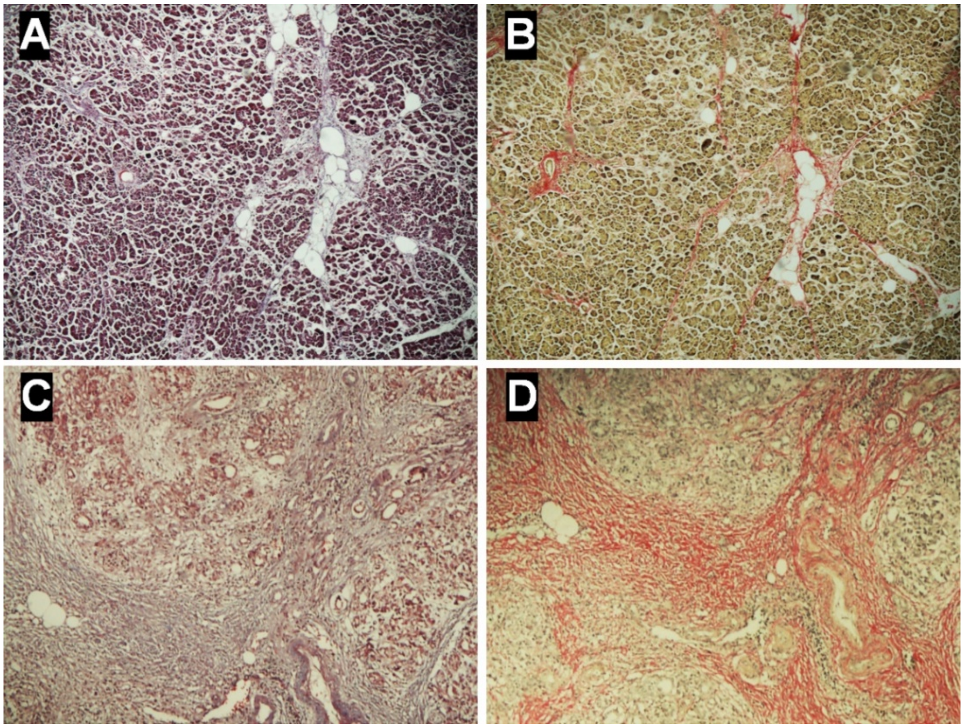

Figure S4. Histology of pancreatic remnants in patient B (A, B) and D (C, D), corresponding with patients in Figure 2. Masson's trichrome and Sirius Red stains in views A & B reveal scant fibrosis (5.7%) of pancreatic remnant (without atrophy), as opposed to more extensive fibrosis (19.7%) and moderate acinar atrophy in views C & D.
